# Ocular Coherence Tomography Unveils Alport Syndrome: A Critical Tool in Detecting Collagen IV Nephropathies

**DOI:** 10.1155/crin/5087883

**Published:** 2024-11-16

**Authors:** Abdelrahman Ibrahim, Zena Altawallbeh, Monica Patricia Revelo, Martin Gregory, Laith Al-Rabadi

**Affiliations:** ^1^Nephrology Department, University of Utah Health, Salt Lake City, Utah, USA; ^2^Pathology Department, University of Utah Health, Salt Lake City, Utah, USA

## Abstract

Collagen IV pathogenic variants are present in Alport syndrome (AS) and some forms of familial focal segmental glomerulosclerosis (FSGS). These conditions pose diagnostic challenges due to overlapping clinical, histological, and genetic features. Ocular coherence tomography (OCT) has emerged as a pivotal diagnostic tool by revealing ocular manifestations characteristic of AS. Here, we present two cases initially diagnosed with primary FSGS but later found to harbor collagen IV pathogenic variants. Both cases progressed to end-stage kidney disease (ESKD) needing transplantation. OCT revealed severe temporal macular thinning consistent with AS in both cases. Our findings highlight the critical role of OCT in distinguishing the subtle differences in the presentation of collagen IV nephropathies. OCT proves valuable for clinicians, particularly when *COL4* nephropathies present ambiguous or overlapping features. In such instances, OCT serves to establish precise diagnoses, preventing unnecessary immune suppression. Therefore, incorporating OCT alongside genetic and histological evaluations is crucial for accurate diagnosis, management, and appropriate genetic counseling. Furthermore, recognizing the prevalence of AS accurately is pivotal for conducting population-based studies, which are essential for advancing our understanding of the condition, improving patient care, and informing future research initiatives.

## 1. Introduction

Collagen type IV is a crucial component of the extracellular matrix, playing an important role in the structure and function of various tissues, including the glomerular basement membranes (GBMs) of the kidneys, eyes, ears, and skin. Pathogenic variants in the *COL4A3, COL4A4*, and *COL4A5* genes lead to collagen IV (*COL4*) nephropathies, including Alport syndrome (AS), thin basement membrane nephropathy, and familial forms of focal segmental glomerulosclerosis (FSGS) [1].

AS primarily affects the GBM and can present with hematuria, proteinuria, and extrarenal manifestations like hearing loss and ocular abnormalities. The disease can be inherited in X-linked, autosomal recessive, or autosomal dominant patterns [2]. FSGS, a common glomerular injury pattern, can result from various causes, including pathogenic variants in *COL4* genes. Though often linked to immune-mediated mechanisms, familial FSGS can overlap clinically with AS due to *COL4* variants [3].

Podocytes, which are integral to the glomerular filtration process, can be injured by certain glomerular diseases [4], including advanced stages of AS [5]. Consequently, AS can result in focal segmental or global glomerulosclerosis in the advanced stages [6].

AS ocular signs include corneal opacities, anterior lenticonus, fleck and dot retinopathy, and temporal retinal macular thinning, which result from the presence of the *COL4 α*3*α*4*α*5 network in the basement membranes of the cornea, lens capsule, and retina. These ocular symptoms are easily identified by simple ocular examination, retinal photography, and ocular coherence tomography (OCT), which is well-known AS diagnostic tools [7].

OCT is a noninvasive, acceptable, cheap, and rapid technique for distinguishing *COL4* nephropathy. In patients with AS, the inner retinal layers sustain changes due to type IV collagen pathogenic variants. While structural changes in each retinal layer have not been fully evaluated, findings from few studies suggest that main alterations occur in the internal limiting membrane and the retinal pigment epithelium basement membrane which is a part of the Bruch's membrane. The Alport retina thinning at the temporal inner and outer macula is likely explained by thinning of the defective ILM as demonstrated on segmentation analysis and electron microscopy [8].

Temporal macular thinning can be measured by using the OCT to calculate the temporal thinning index (TTI). Bilateral temporal thinning is rarely associated with any other systemic conditions [9], so it is important that nephrologists and ophthalmologists be aware of the significance of this finding. Temporal macular thinning has been increasingly recognized as a significant marker in AS. Several studies showed that this thinning is most pronounced in X-linked males, with similarly severe thinning observed in individuals with recessive forms of the disease, affecting both males and females. This finding suggests that retinal temporal thinning could play a role in the diagnosis of AS and in differentiating between various inheritance patterns [10].

In this paper, we describe two cases presenting with proteinuria that were initially diagnosed as primary FSGS. Subsequent genetic testing revealed pathogenic *COL4* variants. OCT studies indicated severe temporal macular thinning in both cases confirming AS.

## 2. Methods

Genetic analysis included DNA from peripheral blood leukocytes, which is processed into libraries, using molecular barcoding and then enriched for coding exons using hybrid capture technology. The pathogenic variants were detected using next-generation sequencing, with copy number variation analysis and confirmed through polymerase chain reaction and Sanger sequencing, Ocular examination was carried out by ophthalmologists using spectral domain OCT in high resolution mode and retinal thickness was assessed by ophthalmologists on Early Treatment Diabetic Retinopathy Study (ETDRS) retinal grids divided into 9 subfields [11].

Assessing the thickness of the temporal retina labeled T1 (inner temporal) and T2 (outer temporal) compared to the nasal retina labeled N1 (inner nasal) and N2 (outer nasal), known as the TTI was calculated using the following formula [12]:

(1) $$\begin{array}{c} \text{TTI }\left(\%\right)=\left\{\frac{\left[\left(N1+N2\right)-\left(T1+T2\right)\right]}{\left(N1+N2\right)}\right\}\times 100. \end{array}$$

Temporal macular thinning in individuals with AS is classified as physiological, moderate, and severe. Severe thinning is defined as TTI exceeding 2 standard deviations above the mean for healthy eyes corresponding to greater than 8.25% in people less than 41 years old and greater than 8.28% in people between 41 and 60 years old [12].

## 3. Case Presentation

### 3.1. Case 1

A 32-year-old female presented with a medical history of hematuria and nephrotic-range proteinuria since childhood.

She has Hashimoto's thyroiditis, treated with levothyroxine, as well as hyperlipidemia and hyperuricemia managed with allopurinol. She was initially diagnosed with immune mediated FSGS and was on steroid therapy for several years. The patient was prescribed lisinopril for proteinuria on her initial visit. Over time, her chronic kidney disease worsened reaching stage V, with estimated glomerular filtration rate (eGFR) up to 13 mL/min. At the age of 30, kidney transplantation was recommended. In preparation for transplantation, she underwent peritoneal dialysis (PD) in the same year. Prior to transplantation, laboratory tests showed a hemoglobin level of 10.1 g/dL, serum creatinine (SCr) concentration of 4.86 mg/dL, and blood urea nitrogen (BUN) of 59 mg/dL. Urinalysis revealed 6 RBC/HPF and an albumin-to-creatinine ratio of 3487 mg/g.

**Figure 1:**
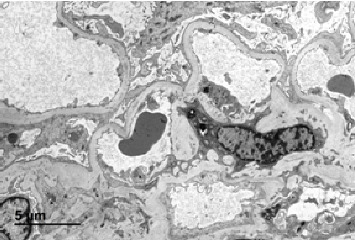
Electron microscopy showing segmental thinning and thickening of the glomerular basement membrane and podocyte effacement.

A kidney biopsy performed at the age of 26 revealed findings consistent with FSGS, along with mild interstitial focal tubular atrophy and occasional aggregates of foamy macrophages in the interstitium. Immunofluorescence staining showed weak staining for IgM and no staining for complement or other immunoglobulins. Electron microscopy revealed segmental thinning of the GBM and segments of GBM thickening but definitive areas of lamellation or basket weaving were not observed. See Figure 1.

An earlier kidney biopsy, taken during the patient's childhood, had shown features consistent with FSGS with foot process effacement. Thin GBM was noted without evidence of basket weave appearance or scalloping.

Genetic testing following the kidney biopsy revealed a novel pathogenic variant, heterozygous for a deletion within the *COL4A5* gene spanning exons 8 and 9. The deletion boundary corresponded to *chrX:107,814,759-107,817,368 (GRCh71/hg19)*. The variant was deemed likely pathogenic, as large intragenic deletions in *COL4A5* have been widely reported to be pathogenic [13].

**Figure 2:**
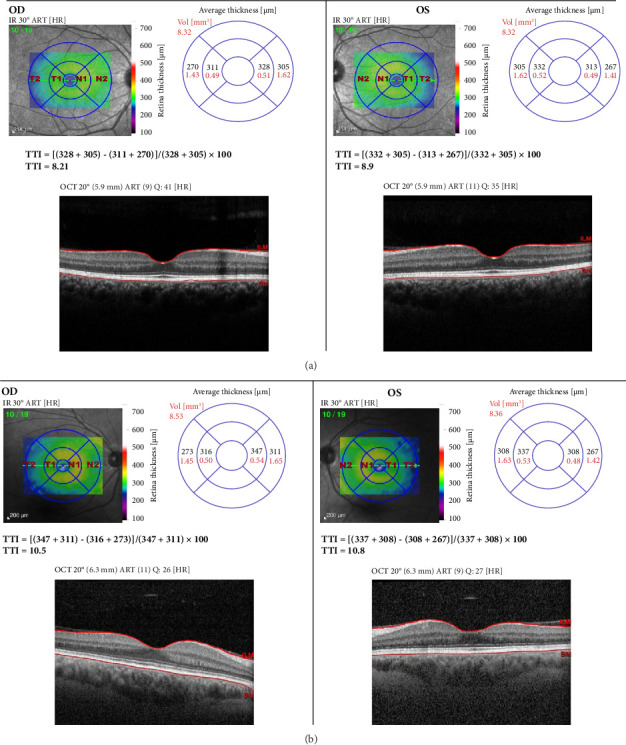
Ocular coherence tomography findings of both patients showing nasal and temporal macular thickness.

At the age of 27, an ophthalmologic examination was done by ophthalmologist. Fundoscopic imaging was adequate with no focal defect. There was no evidence of peripheral or central fleck retinopathy. There were no corneal opacities or erosions. Spectral domain OCT revealed severe temporal macular thinning, with a TTI of 8.21% in the right eye and 8.9% in the left, both values being more than 2 standard deviations for her age group. See Figure 2(a).

The patient has no known family history of kidney disease. After kidney transplantation, she was lost to follow up.

### 3.2. Case 2

A 46-year-old female with a long history of hematuria and proteinuria presented for further management. A kidney biopsy done at the age of 34 revealed FSGS. Initially treated with steroid and cyclosporine therapy for presumed immune-mediated primary FSGS, she later developed steroid resistance. She started lisinopril for proteinuria at the age of 39.

Prior to kidney transplant at the age of 43, laboratory investigations revealed a hemoglobin of 9.3 g/dL. The SCr concentration was 5.19 mg/dL, eGFR dropped to 11 mL/min, and BUN was 66 mg/dL. Urine analysis showed 20 RBC/HPF and total protein/creatinine ratio of 1600 mg/g. She underwent PD 1 year before kidney transplantation.

Genetic testing done at the age of 43 revealed two pathogenic heterozygous variants in the *COL4A3* gene. The first variant was *COL4A3:c.2452G >* *A (p.Gly818Arg)* in exon 31. The second variant, a novel pathogenic variant not previously associated with any clinical condition in the Human Gene Mutation Database (HGMD) and absent from the Broad gnomAD dataset, was *COL4A3:c.4722G > A (p.Trp1574*⁣^∗^), resulting in a stop codon in exon 50.

Ophthalmologic examination done in the same year as genetic testing with spectral domain OCT revealed severe temporal thinning, with a TTI of 10.5% in the right eye and 10.8% in the left eye which is more than 2 standard deviations in this age group. See Figure 2(b). In addition, ocular examinations also revealed posterior subcapsular cataract, but no central perimacular and peripheral coalescing fleck retinopathies. There was no anterior or posterior lenticonus Hearing loss was also reported by the patient since childhood.

The patient has no family history of kidney diseases, and her parents are not currently present for genetic analysis.

## 4. Discussion

In this paper, we described two cases with symptoms of proteinuria as well as histological features characteristic of FSGS, and pathogenic variants in the *COL4* genes. Both cases of AS were definitively diagnosed through genetic testing. The first case revealed a novel heterozygous deletion pathogenic variant in the *COL4A5* gene, while the second revealed a compound heterozygous novel pathogenic variant in the *COL4A3* gene. Both patients developed ESKD with kidney transplantation. There was no reported family history of kidney diseases in either patient. In addition, OCT findings in both patients revealed severe temporal macular thinning, consistent with AS.

In Case 1, the patient exhibited symptoms of AS from childhood and progressed to ESKD by age 30, suggesting a severe phenotype potentially linked to a large deletion pathogenic variant in X-linked AS. Research indicates that proteinuria detected in childhood is associated with rapid progression to kidney failure in females (Gibson et al.) [14]. Moreover, female patients with truncating variants have significantly poorer kidney survival compared to those with other variants, supporting the presence of genotype-phenotype correlations in XLAS (Kim et al.) [15]. In Case 2, despite being female, the patient exhibited a poor prognosis due to autosomal recessive AS. Clinical features in women with this condition are comparable to those in men, with all females eventually progressing to ESKD. There is an early peak in childhood and another around age 30, similar to males with X-linked disease, often accompanied by hearing loss, lenticonus, and retinopathy (Savige et al.) [16]. This connection between severe kidney and ocular phenotypes highlights the importance of considering these relationships in clinical evaluations, as they may reflect underlying genetic severity.

Our manuscript presents two female patients who demonstrate severe retinal thinning, challenging the notion that this finding is predominantly significant in X-linked males. The use of OCT is highlighted as a valuable tool in the diagnosis of AS due to its ease of use, affordability, and noninvasive nature. While those findings can't be generalized to all females (carriers or heterozygous), they can still be helpful and add an important piece of information, especially if positive. OCT can have more role in clinical practice given its availability and convenience compared to more invasive procedures like biopsies. The lack of extensive research on recessive forms of AS suggests that further studies are needed to fully understand the predictive power of OCT in diagnosing *COL4* nephropathies across different inheritance patterns. While these two cases were examined by OCT in ESKD, further studies are needed to determine the utility of OCT in diagnosing AS throughout the progression of the disease.

The overlap between AS and FSGS emphasizes the critical value of genetic and ocular studies for accurate diagnosis. Both conditions can manifest with proteinuria and hematuria [17], but OCT findings which reveal temporal macular thinning favor an AS diagnosis more so than primary FSGS, in reported cases so far [7].

Recent advancements in genetic diagnostics have facilitated the grouping of these diverse conditions under the category of *COL4* nephropathies, thereby emphasizing a common etiology.

Depending on the subtypes of FSGS, various management strategies are considered, with options including the sustained use of steroids in cases of primary FSGS. If steroids are ineffective or not well-tolerated, immunosuppressive medications like calcineurin inhibitors, mycophenolate mofetil, or rituximab are considered, chosen based on the patient's response and the type of FSGS they have. FSGS in patients with nephrotic syndrome carries a risk of progressing to ESKD within 5–10 years. Complete remission reduces this risk significantly, but patients resistant to steroids have a poorer prognosis. Steroid therapy shows a moderate response rate in treating FSGS ranging from 40%–60% [13].

There is no definitive cure for AS. XLAS males face a high risk of progressing to ESKD by age 40, especially with certain genetic pathogenic variants. Females with XLAS have a 40% chance of developing ESKD by age 80. Both males and females with ARAS usually reach ESKD by age 40. ADAS and heterozygous females with XLAS exhibit variable clinical courses, with a significant risk of ESKD if they have proteinuria, FSGS or hearing loss. Therefore, regular nephrology monitoring and consideration of renin-angiotensin system inhibitors are crucial for management [13]. Assessing retinal thinning in patients with ESKD or FSGS of undetermined etiology can help in both the pretransplant evaluation and post-transplant monitoring to assess risk of disease recurrence, known to be unlikely with AS and *COL4* associated-FSGS as opposed to immune mediated.

## 5. Conclusion

To distinguish between various genetic pathogenic variants, OCT was used in both cases to evaluate severe retinal thinning, which has been observed in AS. In summary, OCT can help by detecting *COL4* nephropathy and thus avoiding the administration of immune suppression therapies when they are not needed. OCT represents an effective, noninvasive, cost-efficient, and swift method for differentiating *COL4* nephropathy.

Further studies could help pinpoint the actual prevalence of AS in the population and identify individuals for additional larger cohort studies for understanding conditions related to AS as well as improve upon clinical care and effective therapies for the disease.

In addition, while we acknowledge the limitation of our report, we do anticipate that it will pave the way for more studies that will open avenues for new diagnostic studies to unravel the distinction between those two entities.

## Data Availability

The data that support the findings of this study are available from the corresponding author upon reasonable request.
